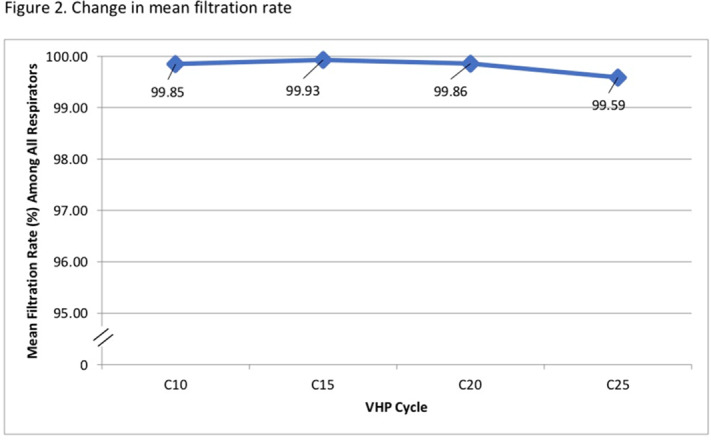# Evaluating N95 Respirator Filtration, Seal, Qualitative and Quantitative Fit with Vaporous Hydrogen Peroxide Reprocessing

**DOI:** 10.1017/ash.2021.38

**Published:** 2021-07-29

**Authors:** Christina Yen, Preeti Mehrotra, Dana Pepe, Sharon Wright, Patrick Gordon, Lalitha Parameswaran

## Abstract

**Background:** The COVID-19 pandemic has created personal protective equipment (PPE) shortages, particularly of N95 respirators. Institutions have used decontamination strategies including vaporous hydrogen peroxide (VHP) to augment respirator supplies. VHP can be used to decontaminate nonporous surfaces without compromising material integrity. However, little is known about its impact on N95 respirator efficacy. We assessed whether repeated VHP reprocessing altered 4 key respirator efficacy qualities: quantitative fit, qualitative fit, seal check, and filtration rate. **Methods:** We conducted a prospective cohort study from June 15 to August 31, 2020. In total, 7 participants were fitted to a 3M 1860 small or regular N95 respirator based on qualitative and quantitative fit testing. Respirators underwent 25 disinfection cycles with the Bioquell BQ-50 VHP generator. After each cycle, participants donned and doffed respirators and performed a seal check. Participants were given 2 attempts to pass their seal check. Every 10 cycles, qualitative fit testing was done using an aerosolized Bitrex solution. Quantitative fit testing was conducted using a PortaCount Pro 8038 Fit Tester to generate a fit factor score. Appropriate fit is defined as a fit factor score of 100 or greater. Quantitative testing was done at cycles 1, 3, 5, 7, 10, 15, 20, and 25. Filtration efficiencies of particles ≥0.3 µm in diameter were measured using the TSI Optical Particle Sizer 3330 at cycles 1, 5, 10, 15, 20, and 25. The Fisher exact test was used to assess qualitative fit and seal check. The Kruskal-Wallis test was used to analyze quantitative fit and filtration rate. **Results:** We observed no seal-check or quantitative-fit test failures during the study window. All participants passed qualitative fit testing. Although there was a significant degree of variability in fit factor scores across disinfection cycles (mean score 163.5, p <0.05), there was no significant difference between participants (p = 0.6) (Figure 1). There was no statistically significant change in mean filtration rate from cycle 10 to 25 (*P* = .05), and the filtration rate remained >95% by cycle 25 (Figure 2). **Conclusions:** VHP reprocessing did not diminish the efficacy of N95 respirators based on the 4 metrics we assessed: filtration rate, seal check, qualitative fit, and quantitative fit. Of significance, the filtration rate remained well above the 95% standard filtration for N95 respirators—even through 25 cycles of reprocessing. VHP reprocessing is a safe, viable strategy to disinfect N95 respirators and extend their use, particularly during supply shortages.

**Funding:** No

**Disclosures:** None

**Figure 1.**
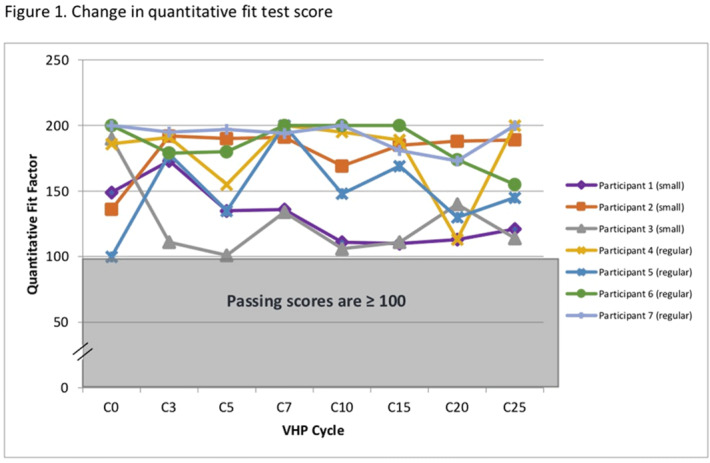

**Figure 2.**